# Open Randomized Clinical Trial on JWSJZ Decoction for the Treatment of ALS Patients

**DOI:** 10.1155/2013/347525

**Published:** 2013-09-05

**Authors:** Weidong Pan, Xiaojing Su, Jie Bao, Jun Wang, Jin Zhu, Dingfang Cai, Li Yu, Hua Zhou

**Affiliations:** ^1^Department of Neurology, Shuguang Hospital Affiliated to Shanghai University of TCM, 528 Zhangheng Road, Pudong New Area, Shanghai 201203, China; ^2^Department of Rehabilitation, First Hospital of Yinchuan, 40 Liqun East Road, Xing Qing Area, Yinchuan 750001, China; ^3^Laboratory of Neurology, Institute of Integrative Medicine, Zhongshan Hospital, Fudan University, 180 Fenglin Road, Shanghai 200032, China; ^4^Department of Cardiology, Shuguang Hospital Affiliated to Shanghai University of TCM, 528 Zhangheng Road, Pudong New Area, Shanghai 201203, China

## Abstract

*Objective*. To investigate the efficacy and safety of the traditional Chinese medicine Jiawei Sijunzi (JWSJZ) decoction for the treatment of patients with amyotrophic lateral sclerosis (ALS). *Methods*. Forty-eight patients with ALS were divided into a JWSJZ group (*n* = 24) and a control group (*n* = 24) using a randomized number method. Together with the basic treatment for ALS, JWSJZ decoction was added to the treatment regimen of patients in the JWSJZ group or Riluzole was administered to the control group for 6 months. Neurologists evaluated the treated and control patients using the ALS functional rating scale (ALSFRS) before, 3 and 6 months after starting the additional treatments. *Results*. The ALSFRS scores in both groups were lower 3 and 6 months after treatment than before. There was a significant difference at 6 months after treatment between the subgroups of patients with ALS whose limbs were the initial site of attack. No serious adverse effects were observed in the JWSJZ group. *Conclusion*. JWSJZ decoction may be a safe treatment for ALS, and may have delayed the development of ALS, especially in the subgroup of patients in whom the limbs were attacked first when compared with Riluzole treatment.

## 1. Introduction

Amyotrophic lateral sclerosis (ALS), also known as Lou Gehrig's disease, is a relatively rare, adult-onset, rapidly progressive, and fatal disease that involves degeneration of spinal cord motor neurons [1]. This disorder causes muscle weakness and atrophy throughout the body, and patients with ALS ultimately lose all voluntary movement. Regardless of the region of onset, however, muscle weakness and atrophy invariably spread to other parts of the body as the disease progresses. Although disease progression varies between individuals, toward the end stages of disease, most patients require ventilator support. Individuals with ALS most commonly die of respiratory failure or pneumonia within 2–5 years of diagnosis. There are no current treatments for ALS. ALS is diagnosed as “flaccidity syndrome” by traditional Chinese theory based on the weakness and atrophy of limbs and body and the fact that most patients are eventually unable to stand or walk, get in or out of bed on their own, use their hands and arms, and have difficulty with chewing, swallowing, and breathing, which ultimately lead to progressive weight loss and increased risk of choking and aspiration pneumonia. Most traditional Chinese doctors believe that the pathogenesis of motor neuron degeneration in ALS has its origin in a deficiency in the spleen, which is the organ that controls the creation of muscle, or deficiency of spleen accompanied by excess expending [2]. “Deal with Yangming meridian alone when treating flaccidity syndrome” is described in the ancient Chinese medicine book Huangdi Neijing [3]. The Yangming meridian means the functions of the spleen. It could be thought that dealing with the spleen alone might improve the flaccidity syndrome. In previous studies, Sijunzi decoction appeared to positively improve and optimize cellular immune function and nutritional status in postsurgical gastric cancer patients [4], as well as improving neuroendocrine regulation in rats with “spleen-deficiency and spleen dysfunction” [5]. Jiawei Sijunzi decoction (JWSJZ) is made from Sijunzi decoction which can nourish the spleen and enrich the vitality of the body, plus the 2 herbs *Radix astragali *and *Desertliving cistanche*. Here we investigated the effects of JWSZJ decoction in the treatment of patients with ALS at 6 months and compared them with those of Riluzole, the only possibly effective “orphan drug” for treating ALS, in an attempt to demonstrate an evidence-based quantitative study of the effects of JWSJZ decoction.

## 2. Subjects and Methods 

### 2.1. Subjects

An open randomized study design was used. Forty-eight patients with probable or definite ALS as defined by the El Escorial criteria [6] diagnosed at the Department of Neurology of Shuguang Hospital Affiliated to Shanghai University of TCM were invited to participate in the study. The age of the patients ranged from 20 to 80 years old $(\overset{-}{x}\pm s,50.4\pm 6.7$ years), and signed informed consent was obtained before participation. The baseline clinical characteristics of the two ALS groups, including age, gender, mean symptom duration at baseline, in months, mean time from diagnosis to baseline in months, and mean ALSFRS scores at baseline are presented in Table 1. The study was approved by The Ethics Committee of Shuguang Hospital Affiliated to Shanghai University of TCM and was performed in accordance with the principles outlined in the Declaration of Helsinki. 

### 2.2. Randomization, Masking, and Drug Administration

An unblinded pharmacist generated randomization codes using an Excel (Microsoft Office) random number generator (Microsoft, USA) in blocks of two and four participants. Kits were given sequential numbers that corresponded to the randomization key and were maintained in a secure location. When randomized, each successive participant was assigned by an electronic clinical trial management system to the next numbered kit in sequence at each site. The ALS patients were randomized into either the JWSJZ group (*n* = 24) or the control group (*n* = 24). There was no stratification of patients according to the onset region, age, or respiratory function since all the patients enrolled were supposed to receive both treatments.

 Patients who had a forced vital capacity of less than 30%, those with signs of a major psychiatric disorder and/or dementia, acute cholecystitis, or bile duct occlusion, or patients who had another concomitant condition thought to be likely to interfere with drug compliance and outcome assessment were excluded. Additional exclusion criteria were pregnancy and participation in other clinical trials. The patients in the JWSJZ group took the JWSJZ decoction (*Panax Jinseng 9 *g, *Radix Astragali 30 *g, *Desert Cistanche 12 *g, *Rhizoma Atractylodis Macrocephalae 9* g, *Poria Cocos 9* g,* Glycyrrhiza 9* g*;* Place all of the herbs into 400 mL of cold water, soak for 30 min, and boil for 30 min using a small flame to obtain about 100 mL of decoction. The decoctions were prepared by the manufacturing laboratory of Shuguang Hospital) (50 mL) twice per day while the other patients were treated with Riluzole tablets (Sanofi-Aventis Co., Ltd., France; 50 mg) twice per day for 6 consecutive months. They were not treated by any other complementary and/or alternative treatments such as other traditional Chinese medicine, Tai Chi exercise, or acupuncture.

### 2.3. Clinical Efficacy and Safety Evaluation

 A detailed history and neurological examination were performed 3 times by a neurologist in all subjects at baseline (before treatment), 3 months, and 6 months throughout the 6-month study period. Disease severity was graded using the revised ALS functional rating scale (ALSFRS) [7]. We used the improvement rate of ALSFRS as the primary result to evaluate the efficacy of the additional treatment. The improvement rates were calculated using the formula (1). Treatment compliance was checked monthly by a pharmacist, and noncompliant patients who took less than 80% of the study medications were considered to have violated the treatment protocol. Changes in the SF-36 physical functioning (PF) subscale [8] and the mean distal limb muscle strength scale were used as secondary results. Standard laboratory tests including red blood cell count, chemistry, renal and liver function, and electrocardiograms were performed at baseline and at the posttreatment discontinuation visit. Safety was evaluated as the incidence and severity of adverse events, and their relationships to treatment were determined based on the results of laboratory tests, patient reports, and the judgment of the investigator.

Consider the following:

(1) $$\begin{array}{c} \text{improvement rate}=\left|\frac{\text{after}-\text{before}}{\text{before}}\right|\times 100\%. \end{array}$$

### 2.4. Statistical Analysis

Repeated-measure ANOVA was conducted to test the differences among changes in outcomes at baseline, at 3 months, and 6 months later for both groups. Differences at baseline between the JWSJZ group and control group were analyzed using the *t*-test. A significant difference was defined as *P* < 0.05. SPSS (Windows version 17.0) software was used for statistical analyses. All data are expressed as the mean ± standard deviation. 

## 3. Results

**Figure 1:**
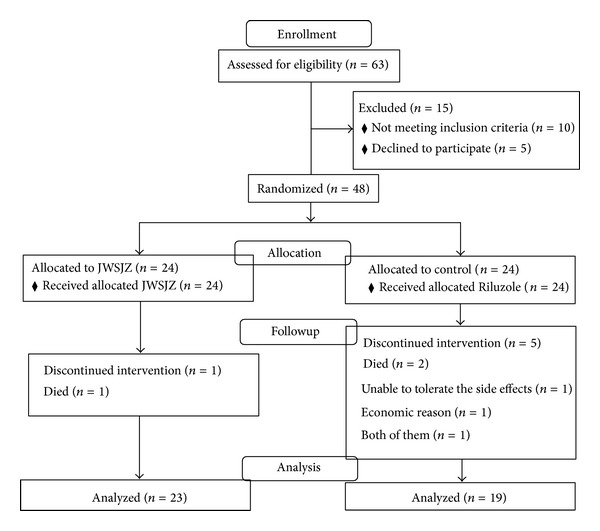
CONSORT flow diagram of JWSJZ decoction for the treatment of patients with ALS.

There were no differences in gender, age, and ALSFRS scores before the additional treatments were started between the two groups (Table 1). In the JWSJZ group, 18 patients first developed ALS in the limbs (defined as subgroup), 3 via bulbar paralysis, and 3 from both syndromes. In the control group, 19 patients first developed ALS in the limbs, 2 via bulbar paralysis, and 3 were attacked by both. Except for the patient who died from development of the disease at the end of the trial, all patients completed the investigation with less side effects (treatment related gastrointestinal side effects: 2 with nausea and 2 with constipation) in the JWSJZ group. No liver/kidney damage was observed in the JWSZJ group. Two patients died from development of the disease, including one limb first attack patient, and 12 patients had drug related gastrointestinal side effects in the control group (including 9 cases with nausea, 6 with dizziness, 7 with anorexia, 3 with constipation, and 3 with diarrhea). Three cases withdrew from the study due to side effects or economic reasons or both in the control group (Figure 1). Four patients had drug related liver/kidney damage.

The changes in ALSFRS score were as follows: the scores of both groups had decreased significantly after 6 months of treatment compared with those before treatment; however, the slope of the decrease in the JWSJZ group was less than that in the control group. No significant differences were found between the endpoint of the two groups (Table 2). The limbs first attack patients (subgroup) in the JWSJZ group had a significantly smaller rate of change (%) of ALSFRS scores after 6 months of treatment compared with the subgroup in the control group (Table 3). The changes in SF-36 physical functioning (PF) sub-scale and muscle strength were as follows: both groups had significant decreases in the PF sub-scale, but there were no significant changes in muscle strength (Table 4) compared with baseline after 6 months of treatment. The PF sub-scale was slightly higher in the JWSJZ group than in the control group at the endpoint, but the difference was not significant. 

## 4. Discussion

Amyotrophic lateral sclerosis (ALS) is a fatal neurodegenerative disorder characterized by progressive degeneration of motor neurons in the motor cortex, brain stem, and spinal cord, leading to paralysis and death, typically within 3–5 years from symptom onset. Riluzole is the only FDA-approved “orphan drug” for ALS and prolongs median survival by only 2-3 months in patients treated for at least 18 months. Importantly, the greatest benefit of Riluzole is observed when treatment is initiated early in the course of the disease, highlighting the importance of early intervention in ALS [9]. Identifying effective treatments for ALS is the most important task for neurologists. Traditional Chinese medicine is one treatment choice for ALS due to its benefits that have been reported in clinical studies [10, 11]. The progressive weakness, muscle atrophy, dysphagia, weight loss, and even respiratory paralysis associated with ALS belong to “flaccidity syndrome or Wei Zheng” in Chinese traditional medicine theory. “Deal with Yangming meridian alone when treating flaccidity syndrome” is one proven theory currently in use for flaccidity treatment. According to traditional Chinese medicine, the functions of the stomach and spleen belong to the Yang Ming meridian. The “Stomach is the reservoir of food and drink” and “the sources of Qi (vitality) and blood manufacture” according to Huangdi Neijing [3], which demonstrates that “Yang Ming is the sea of the viscera internal organs of the body, it can embellish muscle tendons, and the muscle tendons can modify the movement of the joints.” The Yang Ming meridian can manufacture all muscles of the organs and limbs and control their activities. If the Yang Ming is deteriorating, the muscles will atrophy and flaccidity will develop. We based JWSJZ on the famous traditional nourishing spleen and enriching vitality formula, Sijunzu decoction (*Panax ginseng* (Ren Shen), *Rhizoma Atractylodis Macrocephalae *(Bai Zhu), *Poria cocos *(Fu Ling), and *honey-fried licorice root *(Zhi Gan Cao)) [2], plus more potent nourishing spleen and enriching vitality herbs, *Astragalus mongholicus *(Huang Qi) and *Herba cistanche *(Cong Rong), to increase the nourishing functions of the decoction. Previous studies have demonstrated that *Panax ginseng* can improve the immunity of the body [12] and that *Rhizoma Atractylodis Macrocephalae* has neuroprotective effects and can protect against excitotoxicity-induced apoptosis in cultured cerebral cortical neurons [13]. *Poria cocos *and *honey-fried licorice root* have been found to inhibit the development of senile dementia and improve the degeneration of neurons [14]. *Astragalus mongholicus* inhibited high mobility group protein 1-(HMGB1-) induced endothelial cell permeability in endothelial cells and modulates some endothelial functions of the body [15]. The JWSJZ decoction might improve the activity and condition of the muscles, and this may agree with the treatment policy of “Deal with Yangming meridian alone in treating for flaccidity syndrome” when treating ALS. After 6 months of treatment, the severity of the ALS worsened in both groups and the symptoms of the ALS patients were not improved by the JWSJZ decoction, but compared with the Riluzole group, the JWSJZ decoction seemed to be slightly superior at slowing down the speed of development of ALS (Table 1). JWSJZ showed significant effects in the limbs first attacked subgroup patients on reducing development of the disease compared with Riluzole (Table 2). Limbs first attacked patients with limb atrophy and flaccidity syndrome belong to the “flaccidity syndrome or Wei Zheng” and conform more to traditional Chinese medicine theory, while bulbar paralysis first attack patients just suffer a deficiency of vital energy. It is easy to understand why patients whose limbs were attacked first responded more sensitively to treatment with the JWSJZ decoction. No significant differences were observed between the subgroups in the Riluzole group. Furthermore, compared with the Riluzole group, the JWSJZ group demonstrated better dependency, less side effects, and a cheaper price. From 2008 to 2012 in China, the mean price of JWSJZ decoction was about 22.8 yuan/per day, while Riluzole cost was 160 yuan/per day (exchange rate; approximately 6 yuan/US dollar); therefore, the JWSJZ decoction is superior to Riluzole from a price perspective.

Inefficient GluR2 Q/R site editing is a disease-specific molecular dysfunction found in the motor neurons of sporadic ALS patients [16]. Genetically modified mice (designated as AR2) showed a decline in motor function commensurate with the slow death of ADAR2-deficient motor neurons in the spinal cord and cranial motor nerve nuclei [17]. The efficacy of JWSJZ decoction for treating ALS was also observed in vivo using this AR2 mouse model [18]; however, the molecular mechanism is not yet clear and further studies are needed. We have reported the clinical effects of the nourishing spleen and enriching vitality method, JWSJZ decoction, for treating ALS, especially in patients whose limbs were the first point of attack of the disease, compared with the ALS “orphan drug” Riluzole. The traditional Chinese decoction may be one adjuvant treatment for ALS; however, studies with larger sample sizes and/or different dosages will be needed to confirm the efficacy.

## Figures and Tables

**Table 1:** Baseline clinical characteristics of the two ALS groups.

Subjects	JWSJZ group (*n* = 23)	Control group (*n* = 19)	*P* value
Age (years)	51.6 ± 7.2	50.1 ± 4.2	0.89
Men/Women	14/9	11/8	—
Disease duration (months)	25.9 ± 24.7	26.1 ± 24.9	0.37
Mean time from diagnosis (months)	18.35 ± 16.78	17.59 ± 13.51	0.29
Area where ALS first developed—limbs/bulbar/both	18/3/3	19/2/3	—
ALSFRS	37.1 ± 7.6	37.4 ± 9.2	0.65

**Table 2:** Comparison of ALSFRS before and after treatment for JWSJZ group and control group (*χ* ± *S*).

Variable	*n*		ALSFRS			Rate of change (%)
	Before	After	Before	3 m	6 m	
JWSJZ	24	23	38.2 ± 6.3	36.1 ± 8.7	34.4 ± 7.9*	13.57
Control	24	19	37.9 ± 7.7	34.3 ± 6.8	30.6 ± 9.1*	19.26

JWSJZ: Jiawei Sijunzi decoction; ALSFRS: amyotrophic lateral sclerosis functional rating scale; *P < 0.05 compared with before treatment for the same group; rate of change: counted between before treatment and 6 months for the same group.

**Table 3:** Comparison of ALSFRS before and after treatment for limbs first attacked subgroups of JWSJZ group and control group (*χ* ± *S*).

Variable	*n* of limbs first attacked		ALSFRS			Rate of change (%)
	Before	After	Before	3 m	6 m	
JWSZJ	18	18	37.1 ± 7.6	35.2 ± 7.4	33.9 ± 6.2*	10.68^#^
Control	19	18	37.4 ± 9.2	34.5 ± 4.5	31.6 ± 8.9*	19.03

JWSJZ: Jiawei Sijunzi decoction; ALSFRS: amyotrophic lateral sclerosis functional rating scale; *P < 0.05 compared with before treatment for the same group; ^#^ *P* < 0.01, compared with JWSJZ group; rate of change; counted between before treatment and 6 months for the same group.

**Table 4:** Comparison of SF-36 physical function (PF) sub-scale and mean distal limb muscle strength between JWSJZ group and control group before and after treatment (*χ* ± *S*).

Variable	*n*		PF sub-scale			Distal limb muscle strength		
	Before	After	Before	3 m	6 m	Before	3 m	6 m
JWSJZ	24	23	43.1 ± 5.2	42.4 ± 7.3	38.9 ± 4.9*	3.7 ± 0.5	3.7 ± 0.3	3.5 ± 0.7
Control	24	19	42.9 ± 4.9	42.3 ± 6.8	37.6 ± 7.7*	3.7 ± 0.7	3.6 ± 0.6	3.5 ± 0.4

JWSJZ: Jiawei Sijunzi decoction; PF sub-scale: physical function sub-scale; *P < 0.05 compared with before treatment for the same group.

## References

[1] Tandan R, Bradley WG (1985). Amyotrophic lateral sclerosis: part I. Clinical features, pathology, and ethical issues in management. *Annals of Neurology*.

[2] Peng B, Xie J (2007). *Traditional Chinese Internal Medicine*.

[3] Wang B (1979). *The Yellow Emperor's Classic of Internal Medicine—Simple Questions*.

[4] Cai J, Wang H, Zhou S, Wu B, Song H-R, Xuan Z-R (2008). Effect of Sijunzi decoction and enteral nutrition on T-cell subsets and nutritional status in patients with gastric cancer after operation: a randomized controlled trial. *Journal of Chinese Integrative Medicine*.

[5] Liu Q, Cai G (2007). Content of somatostatin and cholecystokinin-8 in hypothalamus and colons in a rat model of spleen-deficiency syndrome. *Journal of Chinese Integrative Medicine*.

[6] Brooks BR, Miller RG, Swash M, Munsat TL (2000). El Escorial revisited: revised criteria for the diagnosis of amyotrophic lateral sclerosis. *Amyotrophic Lateral Sclerosis*.

[7] Cedarbaum JM, Stambler N, Malta E (1999). The ALSFRS-R: a revised ALS functional rating scale that incorporates assessments of respiratory function. *Journal of the Neurological Sciences*.

[8] Ware JE, Sherbourne CD (1992). The MOS 36-item short-form health survey (SF-36). I. Conceptual framework and item selection. *Medical Care*.

[9] Zoing MC, Burke D, Pamphlett R, Kiernan MC (2006). Riluzole therapy for motor neurone disease: an early Australian experience (1996–2002). *Journal of Clinical Neuroscience*.

[10] Wenjie X, Hongli R, Huiping Z (2011). Effects of Kidney-tonifying, Spleen-strengthening and Liver-soothing Method on Amyotrophic Lateral Sclerosis. *Shang Hai Zhong Yi Yao Da Xue Xue Bao*.

[11] Jun W, Junpeng G, Yongmei G (2009). Clinical study of the effect of Fuyuan shengji granule on symptoms of the patients with Amyotrophic Lateral Sclerosis. *Journal of Neurology and Neurorehabilitation*.

[12] Zhai L, Li Y, Wang W, Wang Y, Hu S (2011). Effect of oral administration of ginseng stem-and-leaf saponins (GSLS) on the immune responses to Newcastle disease vaccine in chickens. *Vaccine*.

[13] Gao Q, Ji ZH, Yang Y, Cheng R, Yu XY (2013). Neuroprotective effect of Rhizoma Atractylodis macrocephalae against excitotoxicity-induced apoptosis in cultured cerebral cortical neurons. *Phytotherapy Research*.

[14] Lin Z, Gu J, Xiu J, Mi T, Dong J, Tiwari JK (2012). Traditional Chinese medicine for senile dementia. *Evidence-Based Complementary and Alternative Medicine*.

[15] Zheng YJ, Zhou B, Song ZF (2013). Study of Astragalus mongholicus Polysaccharides on endothelial cells permeability induced by HMGB1. *Carbohydrate Polymers*.

[16] Kawahara Y, Ito K, Sun H, Aizawa H, Kanazawa I, Kwak S (2004). Glutamate receptors: RNA editing and death of motor neurons. *Nature*.

[17] Hideyama T, Yamashita T, Suzuki T (2010). Induced loss of ADAR2 engenders slow death of motor neurons from Q/R site-unedited GluR2. *Journal of Neuroscience*.

[18] Weidong P, Xiaojing S, Jun W (2013). The study for the effects of “Jiawei Sijunzi Decoction” in treating for movement dysfunction of amyotrophic lateral sclerosis AR2 mice. *Shang Hai Zhong Yi Yao Da Xue Xue Bao*.

